# Does the Gadoxetic Acid-Enhanced Liver MRI Impact on the Treatment of Patients with Colorectal Cancer? Comparison Study with ^18^F-FDG PET/CT

**DOI:** 10.1155/2016/8412071

**Published:** 2016-02-28

**Authors:** Ji-Won Oh, Soon Nam Oh, Joon Il Choi, Moon Hyung Choi, Ie Ryung Yoo, Myung Ah Lee, Young-Kyung Yoo, Seong Taek Oh

**Affiliations:** ^1^Department of Radiology, Seoul St. Mary's Hospital, The Catholic University of Korea, 222 Banpo-daero, Seocho-gu, Seoul 06591, Republic of Korea; ^2^Department of Internal Medicine, Seoul St. Mary's Hospital, The Catholic University of Korea, 222 Banpo-daero, Seocho-gu, Seoul 06591, Republic of Korea; ^3^Department of Surgery, Seoul St. Mary's Hospital, The Catholic University of Korea, 222 Banpo-daero, Seocho-gu, Seoul 06591, Republic of Korea

## Abstract

*Objectives*. We evaluated the value of Gadoxetic acid-enhanced liver MRI in the preoperative staging of colorectal cancer and estimated the clinical impact of liver MRI in the management plan of liver metastasis.* Methods*. We identified 108 patients who underwent PET/CT and liver MRI as preoperative evaluation of colorectal cancer, between January 2011 and December 2013. We evaluated the per nodule sensitivity of PET/CT and liver MRI for liver metastasis. Management plan changes were estimated for patients with metastatic nodules newly detected on liver MRI, to assess the clinical impact.* Results*. We enrolled 131 metastatic nodules (mean size 1.6 cm) in 41 patients (mean age 65 years). The per nodule sensitivities of PET/CT and liver MRI were both 100% for nodules measuring 2 cm or larger but were significantly different for nodules measuring less than 2 cm (59.8% and 95.1%, resp., *P* = 0.0001). At least one more metastatic nodule was detected on MRI in 16 patients. Among these, 7 patients indicated changes of management plan after performing MRI.* Conclusions*. Gadoxetic acid-enhanced liver MRI detected more metastatic nodules compared with PET/CT, especially for small (<2 cm) nodules. The newly detected nodules induced management plan change in 43.8% (7/16) of patients.

## 1. Introduction

Colorectal cancer (CRC) is a common cancer in both men and women [1]. Liver metastases are the major cause of death in these patients [2]. Approximately 20–25% of patients with colorectal cancer have liver metastases at the time of diagnosis. Complete resection of liver metastases is the only potentially curative treatment; however, many patients with CRC have unresectable liver metastases [3]. Therefore, an accurate preoperative selection of patients who may benefit from hepatic resection is necessary [4]. The determination of surgical eligibility relies heavily on imaging for the accurate detection of all metastatic nodules [5, 6].

Studies have evaluated the effectiveness of ultrasonography, computed tomography (CT), positron emission tomography (PET), and magnetic resonance imaging (MRI) as preoperative imaging modalities to detect liver metastasis in CRC patient [7–11]. Although the effectiveness of Gadoxetic acid-enhanced liver MRI is recently emphasized [12–15], there still remains the need to set a proper recommendation of liver MRI in preoperative evaluation of CRC.

The 2013 National Comprehensive Cancer Network (NCCN) guideline recommends PET/CT to patients with suspicious lesions on abdominal CT and also mentions that liver MRI can be considered to further evaluate patients diagnosed with potentially resectable hepatic metastasis on CT [16, 17]. PET/CT evaluates peritoneal nodules and other metastatic lesions [18–21]. Liver MRI evaluates suspicious or too small to characterize hepatic lesions detected on abdominal CT [8, 22]. Several reports have mentioned the sensitivity and specificity of PET/CT and liver MRI separately [23, 24]. However, the interpretation of these imaging modalities is not independent in the clinical practice. During the preoperative staging evaluation process, many patients undergo PET/CT first, because liver MRI is usually performed after reviewing the CT results, especially in advanced CRC patients. In terms of cost-effectiveness, it is necessary to evaluate if an additional liver MRI is still required in patients with precedent PET/CT.

To our knowledge, limited data has compared the effectiveness of Gadoxetic acid-enhanced liver MRI with PET/CT to identify liver metastases in CRC patients. This study evaluated the added value of liver MRI in patients with precedent PET/CT, by comparing the detection rate of PET/CT and liver MRI. We also estimated the clinical impact of the additional liver MRI in the management plan of liver metastasis.

## 2. Materials and Methods

### 2.1. Patients

**Figure 1:**
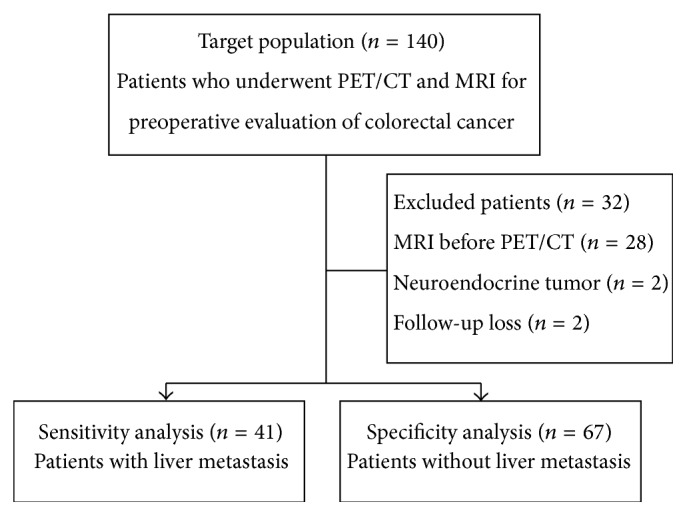
Flowchart of the study population.

Institutional review board approval was obtained, and patient informed consent was waived. We retrospectively reviewed the imaging database of our institute to identify patients who underwent PET/CT and Gadoxetic acid-enhanced liver MRI as preoperative evaluation of colorectal cancer, between January 2011 and December 2013. A total of 140 patients were initially identified. Among them 28 patients with liver MRI performed prior to PET/CT were excluded, because we wanted to evaluate the added value of liver MRI at the clinical setting of already performed PET/CT. Two patients were excluded since the pathologic finding of colorectal lesion was neuroendocrine tumor, and other two patients were excluded because they did not undergo subsequent surgery or follow-up imaging. Finally, a total of 108 patients were enrolled in this study. Patients with at least one metastatic nodule were selected for per patient and per nodule sensitivity analysis. In these patients, liver metastasis was confirmed by subsequent surgery or follow-up imaging. Patients without metastasis were included in per patient specificity analysis.  Figure 1 shows the flowchart of the study population.

### 2.2. Imaging Technique

#### 2.2.1. ^18^F-FDG PET/CT

All patients fasted for at least 6 h before the PET/CT study. Scanning began 60 minutes after the intravenous injection of ^18^F-FDG (370–555 MBq). None of the patients had a blood glucose level greater than 130 mg/dL before the injection. No intravenous contrast agent was administered. Studies were acquired on combined PET/CT inline systems, either Biograph Duo or Biograph TruePoint (Siemens Medical Solutions, Knoxville, TN, USA). The first scan, a whole-body image from the orbitomeatal line to the upper thigh, was performed 1 h after ^18^F-FDG injection. Six to eight bed positions were used and the acquisition time was 2 min per bed position. CT began at the orbitomeatal line and progressed to the proximal thigh (120 kV, 50 mAs, and 5 mm slice thickness; 130 kV, 80 mAs, and 5 mm slice thickness) and was followed by a PET over the same body region. CT data were used for attenuation correction and images were reconstructed using a standard ordered-subset expectation maximization algorithm (OSEM two iterations, eight subsets). Axial spatial resolution was 6.5 or 4.5 mm at the center field of view. All PET/CT images were reviewed with fusion software (syngo; Siemens Medical Solutions, Knoxville, TN, USA) that provided multiplanar reformatted images and displayed PET images with attenuation correction, CT images, and PET/CT fusion images.

#### 2.2.2. Gd-EOB-DTPA-Enhanced Liver MRI

MR imaging was performed using a 3.0-T MR system (MAGNETOM Verio; Siemens Healthcare, Erlangen, Germany) with an eight-channel body phased-array coil. The MR protocol consisted of breath-hold transverse T1-weighted in- and out-of-phase two-dimensional gradient-echo sequences (TR/in phase TE, 130/2.6; out-of-phase TE, 1.5; flip angle, 52°; field of view, 400 × 300 mm; matrix, 288 × 187; section thickness, 6 mm), transverse T2-weighted half Fourier acquisition single shot turbo spin-echo sequences (TR/TE, 800/91; flip angle, 138°; field of view, 400 × 300 mm; matrix, 384 × 173; slice thickness, 6 mm), and transverse fat-suppressed T2-weighted turbo spin-echo sequences (TR/TE, 4400/102; flip angle, 140°; field of view, 400 × 300 mm; matrix, 448 × 218; slice thickness, 6 mm).

A dose of 0.1 mL/kg (0.025 mmol/kg) Gd-EOB-DTPA (Primovist or Eovist, Bayer Schering Pharma) was administered intravenously at 1.0 mL/s for the dynamic study, followed by a 30 mL saline flush. Contrast-enhanced MRI was performed using an axial fat-suppressed three-dimensional T1-weighted spoiled gradient-echo sequence (TR/TE, 3.1/1.2; flip angle, 11.5°; field of view, 380 × 300 mm; matrix, 374 × 200; section thickness, 2 mm). Gd-EOB-DTPA-enhanced hepatocyte-phase images were obtained 20 min after contrast agent administration.

### 2.3. Image Analysis

The clinical reports of PET/CT and Gadoxetic acid-enhanced liver MRI were reviewed for all patients. Two board-certified radiologists reviewed the MR and PET/CT images and recorded the exact location, size, and number of metastatic nodules to assess the accuracy of the initial clinical reports. The maximal diameter of each nodule was measured on the axial image of the hepatocyte-phase, on a single slice in which the size of the lesion was the largest. All nodules were classified as less than 1 cm, 1 cm or larger but less than 2 cm, and 2 cm or larger.

### 2.4. Reference Standard

Final pathologic results were used as the reference standard for lesions of which the operated specimens were available. Suspected lesions on MRI not operated on were followed by repeated imaging studies. A liver metastasis diagnosis was made when a focal hepatic lesion was progressive under therapy, a focal hepatic lesion showed a partial response under therapy, or when a focal hepatic lesion showed a complete response under therapy.

### 2.5. Statistical Analysis

Per patient and per nodule analyses were performed, respectively. Per patient sensitivity and specificity of PET/CT and MRI were calculated and compared, using McNemar's test. Per nodule sensitivities of PET/CT and MRI were calculated and compared for all nodules and among different nodule size, also using McNemar's test. A student *t*-test compared the size of PET/CT negative metastatic nodules and PET/CT positive metastatic nodules.

### 2.6. Assessment of Clinical Impact of Liver MRI

Patients with newly detected metastatic nodule after performing MRI were selected to estimate the clinical impact of liver MRI on the management plan. Virtual management planning was performed for these patients by consensus of a surgeon and a medical oncologist. The first planning was made blind to the MRI results, and the second planning was made after notifying the MRI results. A comparison of these two virtual management plans evaluated significant changes of treatment plan after performing liver MRI.

## 3. Results

Among the initial 108 patients, 41 patients (mean age 65 years; range 37–81) who showed at least one metastatic nodule were selected for per patient sensitivity analysis. Among these, 29 patients were confirmed to have liver metastasis by subsequent surgery and 12 patients were confirmed by follow-up imaging. A total of 131 metastatic nodules (mean size 1.6 cm; range 0.4–8.2) were identified from these 41 patients and included in per nodule sensitivity analysis. Eleven patients had a solitary metastasis, and one patient had a maximum of nine metastases. Sixty-seven patients who had no metastasis were included in per patient specificity analysis.

### 3.1. Per Patient Analysis

Among the 41 patients selected for per patient sensitivity analysis, 27 patients had T3 stage, 14 patients had T4 stage, and no patient had T1 or T2 stage disease. A total of 6 patients had N0 stage, 14 patients had N1 stage, and 21 patients had N2 stage disease.

Per patient sensitivities of PET/CT and MRI were 95.1% (39/41) and 97.6% (40/41), respectively. And per patient specificity of PET/CT and MRI were 100% (67/67) and 92.5% (62/67), respectively. Per patient sensitivity and specificity did not differ significantly (*P* = 1 for sensitivity, and *P* = 0.063 for specificity).

PET/CT showed two false negative patients, whose metastatic nodules were not indicated on the retrospective review. MRI showed one false negative patient with two sub-centimeter subcapsular metastatic nodules identified on the retrospective review.

There were five MR false positive nodules in 5 patients. One patient underwent hepatic resection and the final pathologic diagnosis was lymphoplasma cell infiltration. For other patients, suspicious or undetermined nodules (0.4–1.6 cm) were not operated on; however, follow-up imaging studies revealed nonmetastatic lesions.

### 3.2. Per Nodule Analysis

A total of 131 nodules in 41 patients were included in per nodule sensitivity analysis. Among these, 48 nodules were less than 1 cm, 54 were 1 cm or larger but less than 2 cm, and 29 were 2 cm or larger.

The per nodule sensitivities of PET/CT and MRI were 68.7% (90/131) and 96.2% (126/131), respectively.  Table 1 summarizes the sensitivity of PET/CT and MRI among different size groups. The sensitivities of PET/CT and MRI were significantly different for small nodules measuring less than 2 cm (*P* = 0.0001).

The mean diameter of PET/CT positive metastatic nodules was significantly larger than PET/CT negative metastatic nodules (1.98 cm and 0.75 cm, resp., *P* = 0.0001).

### 3.3. Clinical Impact

**Figure 2:**
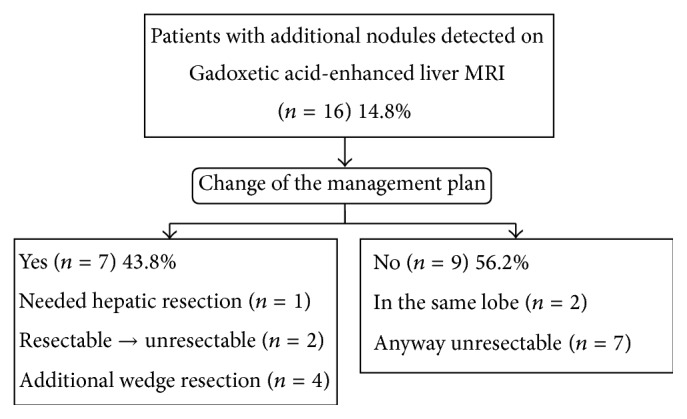
Clinical impact of the Gadoxetic acid-enhanced liver MRI on the management plan of the patients with liver metastasis of colorectal cancer.

**Figure 3:**
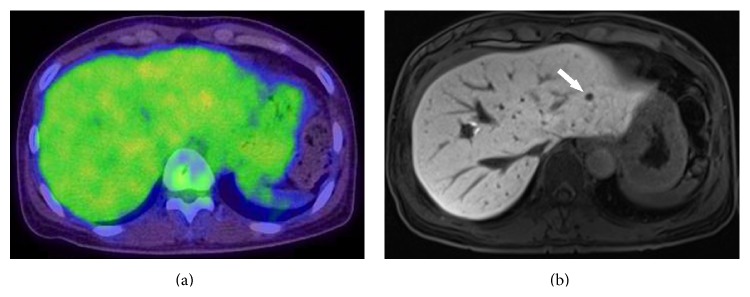
A 51-year-old woman with sigmoid colon cancer, who showed liver metastasis only on Gadoxetic acid-enhanced liver MRI. (a) Axial ^18^F-FDG-PET/CT image shows inhomogeneous physiologic activity of liver but no definite metastatic nodule. (b) Axial image of hepatocyte-phase on liver MRI shows a focal signal defect in segment 2 suggesting metastatic nodule (arrow).

**Figure 4:**
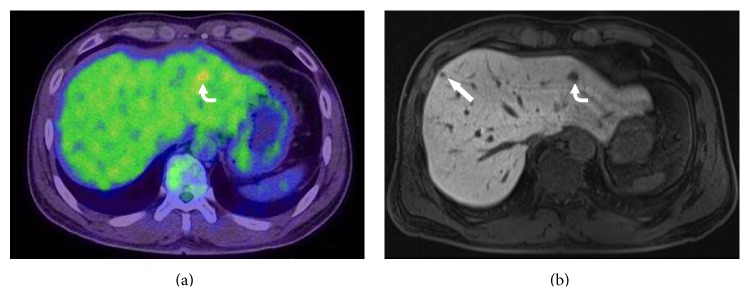
A 46-year-old man with sigmoid colon cancer, who showed one metastatic nodule on PET/CT and two metastatic nodules on liver MRI in both hepatic lobes. (a) Axial ^18^F-FDG-PET/CT image shows a focal increased uptake in left lateral section of the liver (curved arrow), but there is no definite FDG uptake in right hepatic lobe. (b) Axial image of hepatocyte-phase on liver MRI shows focal signal defects in left lateral section of the liver (curved arrow), and also in the subcapsular portion of right hepatic lobe (arrow), suggesting two metastatic nodules in both hepatic lobes.

Thirty-seven nodules negative on PET/CT were newly detected by MRI in 16 patients. Additional nodules were detected in both T3 and T4 diseases, with 10 patients having T3 and 6 patients having T4 disease. According to the virtual management planning, there were no changes of the management plan in 9 patients; however, 7 patients indicated changes of the management plan after performing MRI (Figure 2). One patient needed a hepatic resection for a metastatic nodule only detected by liver MRI (Figure 3). Two patients were reclassified to unresectable liver metastasis and underwent chemotherapy. An additional wedge resection or tumorectomy was needed for 4 patients because newly identified nodules were located in different segments or lobes of liver, with known metastatic nodules (Figure 4).

## 4. Discussion

Colorectal cancer is a malignant tumor in which the presence of limited liver metastasis warrants surgical resection. Effective management depends on the appropriate selection of patients with an accurate preoperative detection of liver metastasis [6]. Many studies have evaluated the effectiveness of various imaging modalities, and liver MRI is known to be the best imaging modality at detecting liver metastasis [7–10, 12, 13, 23–26]. However, the focus of our study was to evaluate if liver MRI would still be helpful for patients with precedent PET/CT in the practical management planning of liver metastasis. These conditions are especially important for patients with advanced CRC, who are likely to undergo both PET/CT and liver MRI. We excluded patients who performed MRI prior to PET/CT, in order to evaluate the additional value of MRI in patients with precedent PET/CT.

According to our study, additional liver MRI performed after PET/CT showed a high sensitivity to detect liver metastasis in the preoperative evaluation of CRC. Subsequently, additional metastatic nodules (not seen on PET/CT) were newly detected in 14.8% (16/108) of all patients who performed liver MRI. There was a prior study performed by Muhi et al. that also reported a high sensitivity of liver MRI to detect tiny metastatic nodules [9]. A high sensitivity of liver MRI to detect metastatic nodules might be meaningless without a significant change in the management plan after performing liver MRI; therefore, we evaluated the clinical impact of liver MRI regarding the change of management planning before and after liver MRI. In our study, 43.8% (7/16) of patients with newly detected metastatic nodules needed to change the management plan.

Considering the size of nodules, the sensitivities of both liver MRI and PET/CT were 100% for metastatic nodules with a diameter of 2 cm or larger. However, the sensitivity of the liver MRI was significantly higher for smaller nodules due to the markedly decreased sensitivity of PET/CT. The sensitivity of PET/CT for less than 1 cm sized metastatic nodules was only 27.1% (13/48). Coenegrachts et al. reported similar drop of PET/CT sensitivity with the decreasing size of the hepatic nodules [24]. The reduced sensitivity of PET/CT to detect small metastatic nodule is associated with poor spatial resolution.  Table 1 indicates that 11.1% of nodules measuring 1 cm or larger but less than 2 cm, and 72.9% of nodules less than 1 cm were not detected on PET/CT; consequently, we recommend liver MRI as a decision tool, for small suspicious nodules less than 2 cm, even after performing PET/CT. Our study results would be helpful in the determination of the need of liver MRI.

There are several limitations to this study. First, it was a retrospective study, with a relatively small sample size. Second, there was a selection bias in our patient group. Only advanced CRC patients were included, because PET/CT was usually performed in such patients; consequently, result of our study may not be applicable for early stage CRC patients. Finally, MR sensitivity might be overestimated because MR images were interpreted after PET/CT. However, this overestimation may not be significant, since the sensitivity of PET/CT is much lower than liver MRI.

## 5. Conclusions

In conclusion, the added Gadoxetic acid-enhanced liver MRI in advanced stage CRC patients with precedent PET/CT showed a significantly higher sensitivity to detect small metastatic nodules that resulted in additional detection of liver metastasis in 14.8% (16/108) of patients. The additional detection of liver metastasis led to a change of management plan in 43.8% (7/16) of patients; therefore, we conclude that the Gadoxetic acid-enhanced liver MRI should be recommended in advanced CRC patients even after performing PET/CT, to evaluate hepatic nodules less than 2 cm. However, a larger study with similar design that includes early stage CRC patients is required to apply this recommendation to all CRC patients.

## Figures and Tables

**Table 1:** Per nodule sensitivity (%) of PET/CT and Gadoxetic acid-enhanced liver MRI, at detecting metastatic liver nodules in colorectal cancer patients.

	PET/CT	MRI	*P* value
All (*n* = 131)	68.7 (90/131)	96.2 (126/131)	0.0001

≥2 cm (*n* = 29)	100 (29/29)	100 (29/29)	1

<2 cm (*n* = 102)	59.8 (61/102)	95.1 (97/102)	0.0001
≥1 cm, <2 cm (*n* = 54)	88.9 (48/54)	98.1 (53/54)	0.125
<1 cm (*n* = 48)	27.1 (13/48)	91.7 (44/48)	0.0001
